# Transcriptomic analyses to summarize gene expression patterns that occur during leaf initiation of Chinese cabbage

**DOI:** 10.1093/hr/uhae059

**Published:** 2024-02-28

**Authors:** XiaoXue Sun, Zihan Liu, Rui Liu, Johan Bucher, Jianjun Zhao, Richard G F Visser, Guusje Bonnema

**Affiliations:** State Key Laboratory of North China Crop Improvement and Regulation, Key Laboratory of Vegetable Germplasm Innovation and Utilization of Hebei, Ministry of Education of China-Hebei Province Joint Innovation Center for Efficient Green Vegetable Industry, College of Horticulture, Hebei Agricultural University, Baoding 071000, China; Plant Breeding, Wageningen University & Research, 6708 PB Wageningen, The Netherlands; State Key Laboratory of North China Crop Improvement and Regulation, Key Laboratory of Vegetable Germplasm Innovation and Utilization of Hebei, Ministry of Education of China-Hebei Province Joint Innovation Center for Efficient Green Vegetable Industry, College of Horticulture, Hebei Agricultural University, Baoding 071000, China; Plant Breeding, Wageningen University & Research, 6708 PB Wageningen, The Netherlands; State Key Laboratory of North China Crop Improvement and Regulation, Key Laboratory of Vegetable Germplasm Innovation and Utilization of Hebei, Ministry of Education of China-Hebei Province Joint Innovation Center for Efficient Green Vegetable Industry, College of Horticulture, Hebei Agricultural University, Baoding 071000, China; Plant Breeding, Wageningen University & Research, 6708 PB Wageningen, The Netherlands; Plant Breeding, Wageningen University & Research, 6708 PB Wageningen, The Netherlands

## Abstract

In Chinese cabbage, rosette leaves expose their adaxial side to the light converting light energy into chemical energy, acting as a source for the growth of the leafy head. In the leafy head, the outer heading leaves expose their abaxial side to the light while the inner leaves are shielded from the light and have become a sink organ of the growing Chinese cabbage plant. Interestingly, variation in several ad/abaxial polarity genes is associated with the typical leafy head morphotype. The initiation of leaf primordia and the establishment of leaf ad/abaxial polarity are essential steps in the initiation of marginal meristem activity leading to leaf formation. Understanding the molecular genetic mechanisms of leaf primordia formation, polar differentiation, and leaf expansion is thus relevant to understand leafy head formation. As *Brassica*'s are mesa-hexaploids, many genes have multiple paralogues, complicating analysis of the genetic regulation of leaf development. In this study, we used laser dissection of Chinese cabbage leaf primordia and the shoot apical meristem (SAM) to compare gene expression profiles between both adaxial and abaxial sides and the SAM aiming to capture transcriptome changes underlying leaf primordia development. We highlight genes with roles in hormone pathways and transcription factors. We also assessed gene expression gradients along expanded leaf blades from the same plants to analyze regulatory links between SAM, leaf primordia and the expanding rosette leaf. The catalogue of differentially expressed genes provides insights in gene expression patterns involved in leaf development and form a starting point to unravel leafy head formation.

## 1. Introduction

In plants, leaves are the principal organs for photosynthesis, converting sunlight energy into chemical energy [1]. Light capture by the leaves is affected by leaf shape, size, and angle and depends on many aspects, such as leaf position, environmental gradients and genetic predisposition [2, 3]. In the model plant *Arabidopsis*, leaf development starts from the shoot apical meristem (SAM) and includes leaf initiation, abaxial/adaxial differentiation, vascular development, cell division and expansion [4]. The differentiation of cells of the SAM into leaf primordia is governed by the local accumulation of the plant hormone auxin [5]. After acquiring ‘leaf’ identity, leaf growth is sustained by the development of successive polarity gradients. Although leaf initiation is not yet fully understood at the molecular level, many genes have been identified with roles in the regulatory networks. Meanwhile, the classical plant hormones auxin, ethylene (ET), gibberellins (GAs), abscisic acid (ABA), cytokinin (CK), and the brassinosteroids (BRs) all contribute to leaf development [6]. Genes involved in phytohormone biosynthesis, transport or signaling also exert their influence on the determination of the leaf polarity and leaf blade expansion and shape.

In the *Arabidopsis* SAM, the homeobox family of transcription factors *KNOTTED1-like HOMEOBOX* (*KNOX*) genes comprising *SHOOT MERISTEMLESS* (*STM*), *BREVIPEDICELLUS* (*BP*), *KNOTTED-LIKE FROM ARABIDOPSIS THALIANA 2* (*KNAT2*)*,* and *KNOTTED-LIKE FROM ARABIDOPSIS THALIANA 6* (*KNAT6*) are expressed and distinguish leaf founder cells from meristem cell fate cells [7, 8]. In particular, the repression of the *KNOXI* gene *BP* coordinates the auxin accumulation that results from PIN1-mediated polar transport, to determine the leaf primordium position [9]. Other hormones, like GA and CK are also regulated by *KNOX* transcription factors and the downregulation of *KNOX* genes leads to a low CK to high GA ratio promoting leaf initiation [10]. In addition, the downregulation of *KNOXI* is maintained by actin-related protein (*ARP*) genes *AS1* and *AS2*, which display a mutually exclusive expression pattern during early leaf development [11]. After leaf primordium initiation, the establishment of adaxial-abaxial polarity is important for the subsequent asymmetric growth of the leaf and expansion of the blade [12]. In *Arabidopsis*, members of the *class III homeodomain-leucine zipper* (*HD-ZIP III*), *KANADI* (*KAN*), *auxin response factors 3/4* (*ARF3/4*), and the *YABBY* gene family (*YAB*) are required in leaf adaxial-abaxial polarity establishment. The *AS1*/*AS2* complex positively regulates *HD-ZIPIII* through reducing *miR165*/*166* expression and directly negatively regulates *KAN* and *YAB* in leaf primordia [13, 14]. *HD-ZIP III* genes *REVOLUTA* (*REV*), *PHAVOLUTA* (*PHV*), and *PHABULOSA* (*PHB*) are expressed in the leaf adaxial side and are inhibited by *KANs* (*KAN1, KAN2,* and *KAN3*) that display a similar expression pattern in abaxial position and promote abaxial cell fate [15]. As with the *KANs*, *YAB1*, *YAB2*, *YAB3,* and *ARF3/4* are expressed on the abaxial side and contribute to the specification of abaxial identity. *KANs* and *YABBY*, *YABBY,* and *ARF3/4* establish a positive feedback loop on the abaxial side [4]. Because *ARFs* regulate the expression of auxin responsive genes, the local auxin gradients along the abaxial/adaxial axis contributes to the leaf polarity formation [16]. Auxin acts as polarizing cue through control of PIN polar targeting by the Aux/IAA-ARF-dependent pathway [17]. Several recent studies found that simple change of the expression of leaf polarity-related genes affects the final leaf shape [18, 19]. Interestingly, the cup-shaped trap formation and growth from planar leaves in *Utricularia gibba* (*Lentibulariaceae*) can be prevented by induction of *UgPHV1* expression on the adaxial side of the leaf [19]. These regulatory mechanisms are not well understood in *Brassica* species, which share a common ancestor with *Arabidopsis thaliana*, but evolved after a whole genome triplication (WGT) event resulting in multiple copies of many genes including those described above important for leaf shape [20].

Interestingly, variation in leaf size and shapes of *Brassica* crop species is among others characterized by its often strong leaf curvature. Both cabbages and heading Chinese cabbage have large upwards curving rosette leaves and inwards curving heading leaves with midveins that broaden towards the leaf base [21, 22]. The inner leaves of the leafy head are shielded from sunlight, while for the outer leaves of the leafy head, the abaxial side is exposed to the light, while the adaxial side is oriented towards the head. The process of leafy head formation is still not fully understood; however, mutations in genes involved in the ad/abaxial polarity pathways are associated with the leafy head phenotypes of both cabbages (*Brassica oleracea* ssp *capitata*) and Chinese cabbages (*Brassica rapa* ssp *pekinensis*) [20]. As these genes have often several paralogues, the associated genetic networks for leaf growth and development in *Brassica*'s likely differ in complexity from those in *Arabidopsis*.

Here, we observed the cellular organization of both mature rosette leaves, the shoot apical meristem (SAM) and leaf primordia around the SAM in heading Chinese cabbage at rosette stage. In addition, we profiled gene expression from laser dissected cells from both the SAM and adaxial/abaxial sides of leaf primordia and along gradients of expanded rosette leaves. We analyzed the transcriptional network that is associated with leaf initiation, polarity and expansion and further investigated transcription abundance of genes and their paralogues with special focus on genes involved in ad/abaxial polarity formation, hormonal regulation and transcription factors. These analyses provide knowledge of genes involved in leaf primordia and polarity formation and rosette leaf growth, which sheds light on Chinese cabbage development and domestication.

## 2. Results

### 2.1. Gene expression in Chinese cabbage leaf primordia and rosette leaves

**Figure 1:**
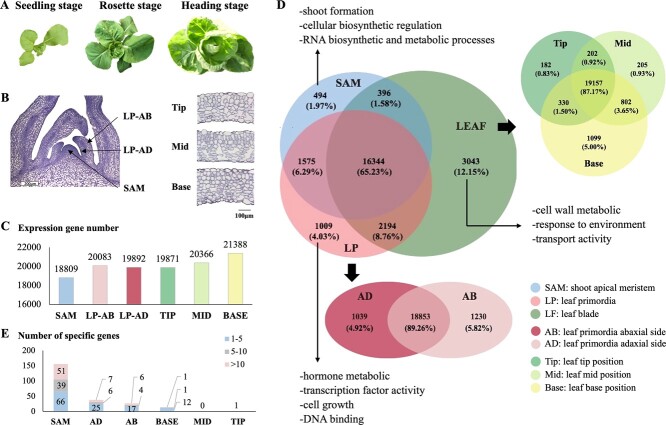
Phenotype of heading Chinese cabbage Z16 and distribution of gene expression. (A) Developmental stages of Z16, including seedling, rosette and heading stages. (B) Transverse sections of the shoot apical meristem (SAM), young leaf primordia (LP: adaxial and abaxial sides), expanding rosette leaf (LEAF: base, mid and tip of rosette leaf). (C) Number of genes expressed in SAM, LP-AD, LP-AD, LEAF-Tip, LEAF-Mid, and LEAF-Base. The expressed genes have FPKM value >1. (D) Distribution of genes expressed and GO terms analysis for specific genes in each tissue. (E) Number of tissue specific genes in SAM, AD, AB, LF-B, LF-M, and LF-T with their transcript abundance levels expressed in FPKM values. FPKM).

Heading Chinese cabbage Z16 development starts from seedling stage, followed by rosette stage and finally the formation of a leafy head at heading stage (Figure 1A). The seedling leaves are round with long petioles, the rosette leaves differentiate by growing larger with short petioles and up-wards curving blades and the heading leaves are upward and inward curving, tightly folding around the shoot apex forming a leafy head. At the rosette stage (4 weeks after transplanting), the expanding rosette leaves display upward growth and early leaf incurvature. Cytological observation reveals the typical dorsiventral structure, palisade cells at the adaxial side and spongy parenchyma cells at the abaxial side. In Z16, the palisade and spongy mesophyll cells are clearly differentiated at both the tip and middle position of the leaf, but not at the base of the leaves that was not completely adaxialized, as palisade parenchyma cells were not formed and the arrangement of the abaxial cells was even with little air space. At the same time, the cells in the first leaf primordia surrounding the SAM are not yet fully differentiated; adaxial and abaxial sides could, however, be distinguished, flanking the more densely packed middle layer of cells, despite the fact that the differentiation of palisade and spongy tissues was not yet visible (Figure 1B).

We generated amplified RNA from shoot apical meristem (SAM) and leaf primordia (LP) (AD-adaxial side and AB-abaxial side) cell populations, isolated from cells dissected by LCM from plants six weeks after sowing. Simultaneously with the preparation of the SAM and leaf primordial tissue, we also harvested tissue from expanding rosette leaves (LEAF) (B-base, M-mid and T-tip) for RNA isolation from the same plant. With the cutoff of 1 fragments per kilobase per million mapped fragments (FPKM value), we detected 18 809 genes expressed in SAM, 21122 genes in LP, including 20 083 genes in LP-AB, 19892 genes in LP-AD and 21 977 genes in LEAF (Figure 1C). Cell type specific transcriptome analysis of leaf primordia revealed that approximately 18 853 (89%) genes were expressed in both AD and AB, whereas 6% were AB cell specific and 5% were AD cell specific (Figure 1D). In expanding rosette leaves, gene numbers expressed in different leaf positions slightly decline from base to tip: base (B: 21388) > mid (M: 20366) > tip (T: 19871). We found that out of the total 25 055 expressed genes, the majority (16 344–65.23%) was shared in both SAM, leaf primordia and rosette leaves. In contrast, only relatively few genes were specifically active in a single developmental stage, including 3034 in LEAF, 494 in SAM and 1009 in LP with cutoff value of 1 FPKM. GO enrichment analysis of these genes revealed shoot formation, cellular biosynthetic regulation, RNA biosynthetic and metabolic processes terms enriched in the SAM, hormone metabolic, transcription factor activity, cell growth, DNA binding terms in LP and cell wall metabolic, response to environment, transport activity terms in LEAF.

### 2.2. Uniquely expressed specific genes during leaf development

Altogether 1708 genes, including 156 in SAM, 27 in AB, 38 in AD, 14 in LEAF-B and 1 in LEAF-T, but none in LEAF-M, were only expressed in a single tissue or position with cutoff value of 1 FPKM (Table S1). Most genes had ‘low’ (1–5 FPKM), while few of these uniquely expressed genes had ‘high’ (>10 FPKM) transcript abundance levels, especially genes in SAM with high transcript abundance levels (Figure 1E).

The genes with unique ‘high’ expression will be referred to as “marker genes” across the SAM, AD, AB tissues and leaf positions. In SAM, 51 marker genes included many transcription factors, such as homeobox transcription factors *WUS1* (*BraA06g030010.3C*), *KNAT6* (*BraA08g026520.3C*), *KNAT1* (*BraA03g026450.3C*), *WOX3* (*BraA04g020450.3C*), *FRU* (*BraA04g020140.3C*), *WRKY* transcription factors *WRKY13* (*BraA08g022420.3C*) and *MADS* box transcription factor *AGL8* (*BraA03g043880.3C*). In addition, SAM maintenance genes *UFO (BraA08g010320.3C), CUC2 (BraA02g014280.3C)* and some not assigned genes were also included. In leaf primordia and the base leaf position, only few genes were in the “marker gene” class, including 6 genes in AB (including *NF-YB5* and *BraA04g026520.3C*, *BraA08g010970.3C*, *BraA09g039320.3C*, *BraA09g041300.3C*, *BraA09g044450.3C*), 7 genes in AD (including *MYB105* and *BraA01g003820.3C*, *BraA01g003830.3C*, *BraA05g032820.3C*, *BraA03g021330.3C*, *BraA02g039160.3C*, *BraA10g031400.3C*) and 1 gene in LEAF-B (*BraA03g012280.3C*). Note that many of these genes have unknown functions in both *B. rapa* and *A.thaliana* databases. Gene expression in leaf positions M and T was very similar, reducing the marker genes for these positions. For that reason, we also defined marker genes ignoring the middle position. When doing so, the number of uniquely expressed genes distinguishing Tip and Base increased from 1 to 16 (Tip) and 14 to 55 (Base), see Table S1.

### 2.3. Differential expression in leaf primordia

The analysis of transcript accumulation within the micro-dissected tissues resulted in 6119 differentially expressed (DE) genes (up: 4096, down: 2023) comparing AD to SAM, 6510 DE genes (up: 4472, down: 2038) comparing AB to SAM, and 1457 DE genes (up: 823, down: 634) comparing AB to AD, with *P*-adjust <0.05 and |log_2_FC| > = 2 (Table S2).

#### 2.3.1. Hormonal regulation, an important regulatory process in leaf primordia

**Figure 2:**
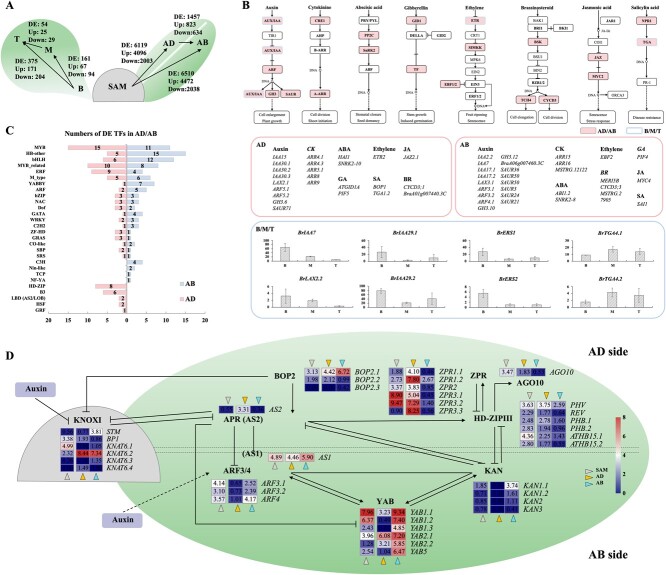
Laser microdissection of leaf primordial domains. (A) Number of DEGs in pairwisecomparisons between SAM, LP domains, and leaf (B) DEGs involved in plant hormone signal transduction pathway and relative expression of DEGs in auxin signal transduction pathways in SAM, AD, AB, and rosette leaves. Color filled boxes indicate the DEGs between AD and AB. Highlight edges indicate the DEGs between leaf positions of the rosette leaves. Of (C) Distribution of differential expressed TF families between adaxial and abaxial side of leaf primordia. (D) Relative expression in SAM, AD, and AB tissues of transcripts associated with the leaf polarity formation.

To identify the biological pathways that are activated in leaf primordia, the DEGs were mapped into the KEGG database record of the pathways. For AD and AB comparison in leaf primordia, we found as TOP1 significantly enriched “plant hormone signal transduction pathway” (pathway ID: map04075), and this pathway was also significantly enriched (TOP 3) in the comparisons between meristem and leaf primordia (*P* < 0.05). In total 166 DEGs were involved in hormone signal transduction pathways: auxin (71 DEGs), cytokinine (20 DEGs), abscisic acid (20 DEGs), gibberellin acid (6 DEGs), ethylene (7 DEGs), brassinosteroid (15 DEGs), jasmonic acid (15 DEGs), and salicylic acid (12 DEGs) (Figure 2B; Table S2).

In the auxin transduction pathway, 27 members of the *AUXIN/IAA* family, 12 members of the *glycoside hydrolase 3* family (*GH3*), 27 members of the *small auxin-up RNA* family (*SAUR*) and 13 members of the *auxin response factor family* (*ARF*) were differently expressed between SAM and leaf primordia AD or AB cells. Among them, some genes from the same family have often opposite expression patterns in leaf primordia, either highly expressed in AD or AB (Figure 2B). In *AUXIN/IAA* family, 5 *AUXIN/IAA* genes (*BrLAX3.1, BrIAA2.2, BrIAA7, BrIAA17.1, BrIAA17.2*) were highly expressed in AB and 5 *AUXIN/IAA* genes (*BrLAX2.1, BrIAA15, BrIAA30.1, BrIAA30.2, BrIAA30.3*) were highly expressed in AD. In *ARF* family, *BrARF3.1, BrARF3.2, BrARF4* were highly expressed in AB, while *BrARF5.1*, *BrARF5.2* were lowly expressed in AB. Other hormone signal transduction pathways were also represented in the DEGs between adaxial and abaxial sides during leaf outgrowth: *ARR-A* (*BrARR4.1, BrARR4.3, BrARR5.1*, *BrARR8, BrARR9*, *BrARR15* and *BrARR16*) in CK, *PIF* (*BrPIF4, BrPIF5*) in GA, auxin and BR, *CYCD3* (*BrCYCD3;1, BrCYCD3;3*) in BR, *NRR1* (*BrBOP1, BrSAI1*) in SA, *ABI1.2* and *HAI1* in ABA pathways. In contrast, few plant hormone signal transduction genes had different expression patterns in different LEAF positions. *BrIAA7*, *BrLAX2.2*, *BrIAA29.1*, *BrIAA29.2*, *BrERS1*, and *BrERS2* were highly expressed in base, while *BrTGA4.1* and *BrTGA42* were low expressed in base, but highly expressed in mid and tip of rosette leaves.

#### 2.3.2. Transcription factors in developing leaf primordia

Leaves have a clear tissue differentiation between upper and lower side: adaxial palisade parenchyma and abaxial spongy parenchyma. In leaf primordia, these parenchyma tissues are not yet visible, but the adaxial and abaxial domains are already established and a number of transcription factors have been shown to participate in the leaf adaxial/abaxial polarity formation. Of the 1457 DEGs between AD and AB, 823 DEGs were higher expressed in the AB side and 634 DEGs were higher expressed in the AD side.

In leaf primordia, we found in AD 1013 transcription factors (TFs) and in AB 1029 TFs expressed. Among them, 195 TFs were detected as DEGs between ad- and abaxial cells in leaf primordia: 100 DEGs were higher expressed in AB side and 95 DEGs were higher expressed in AD side (Table S2). Twenty-eight TF families had different AD/AB expression patterns, including *MYB*, *HD-other*, *bHLH*, *MYB related*, *ERF, M type*, *YABBY*, *ARF*, *bZIP*, and others (Figure 2C). In particular, TFs of *C3H* (n = 4: *BraA03g017500.3C*, *BraA07g030850.3C*, *BraA10g003570.3C*, and *BraAnng001760.3C*), *Nin-like* (n = 2: *BraA01g003130.3C* and *BraA06g016120.3C*), *TCP* (n = 1: *BraA07g030260.3C*) and *NF-YA* (n = 1: *BraA10g029200.3C*) families were only highly expressed in AB side, while TFs of *HD-ZIP* (n = 8: *PHV*, *REV.1*, *PHB.1*, *PHB.2*, *ATHB15.1*, *ATHB15.2*, *HDG4*, and *HDG9*), *B3* (n = 6: *EDF1*, *EDF3*, *REM16*, *REM22*, *BraA07g013190.3C* and *BraA09g009240.3C)*, *LBD* (n = 2: *AS2* and *LBD25*), *HSF* (n = 2: *BraA03g054150.3C* and *BraA10g006890.3C*), and *GRF* (n = 1: *GRF6*) families were all highly expressed in AD side.

Studies in *Arabidopsis* indicated that genes involved in establishing leaf polarity include *HD-ZIPIII, KANADI, ARF*, and *YABBY* transcription factors [23]. Due to the whole genome triplication (WGT) event, many *Arabidopsis* leaf polarity related genes have multiple copies in *B. rapa* (Table S2). Figure 2D provides an overview of expression levels of well-known TFs involved in leaf polarity formation in AD or AB cells of leaf primordia and the meristem. In SAM, the class I KNOTTED-like homeodomain transcription factors (KNOXI) are expressed. Compared to the leaf primordia, *BrSTM, BrKNAT6.2*, *BrKNAT6.3,* and *BrKNAT6.4* expression is low in SAM but high in the leaf primordia. In addition, in AB side, members of auxin response factors (*ARF*: *BrARF3.1*, *BrARF3.2*, *BrARF4.1*), *YABBY* factors (*YAB*: *BrYAB1.1*, *BrYAB1.2*, *BrYAB1.3*, *BrYAB2.1*, *BrYAB2.2*, and *BrYAB5*), and *MYB-*related family transcription factors (*BrKAN1.1*, *BrKAN1.2*, *BrKAN2.1,* and *BrKAN3.1*) were highly expressed. In AD side, eight Class III Homeodomain Leucine Zipper transcription factors (*HD-ZIPIII*: *BrPHV, BrREV.1, BrPHB.1, BrPHB.2, BrATHB15.1,* and *BrATHB15.2*) were highly expressed. The *MYB* related transcription factor *ASYMMETRIC LEAVES 1* (*BrAS1.1*) and the lateral organ boundaries domain transcription factor *ASYMMETRIC LEAVES 2* (*BrAS2*), involved in the *AS1-AS2* pathway to promote leaf adaxial fate, have opposite expression patterns. *BrAS1* was highly expressed in AB and *BrAS2* highly expressed in AD. Besides these TFs, other genes related to leaf polarity formation, for example *AGO10* and *ZPR*, including *BrAGO10.1*, *BrZPR1.1, BrZPR1.2, BrZPR2.1, BrZPR3.1, BrZPR3.2,* and *BrZPR3.3*, which form a feedback loop with HD-ZIPIII, were highly expressed in AD side. Some paralogues of the above genes were not differentially expressed between AD and AB sides, such as *BrARF4.2, BrYAB2.3, BrKAN1.3, BrKAN2.2, BrKAN3.2, BrREV.2, BrZPR2.2, AGO10.2,* and *BrAS1.2*. We also analysed which of the 195 TF genes were co-expressed with these well-known leaf ad/abaxial polarity genes based on their expression in all tissues (SAM, AD/AB, and LEAF) (Table S3).

#### 2.3.3. Gene expression trends along the leaf blade

**Figure 3:**
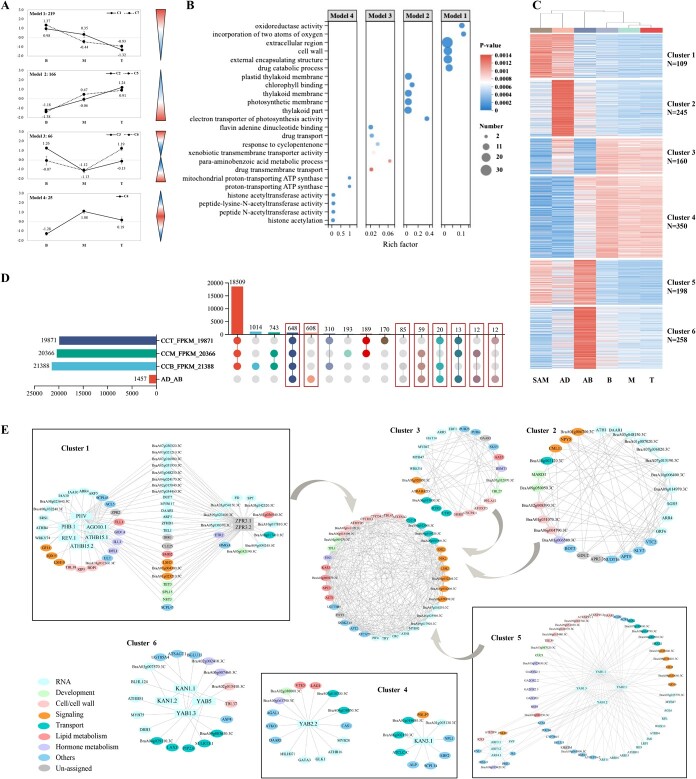
Dynamic progression of DEGs in Chinese cabbage CC-Z16 leaf and leaf primordia transcriptome. (A) K-means clustering showing the expression profile of the 476 DEGs along the three LEAF developmental zones (base, mid and tip). (B) GO analysis of 476 DEGs in four expression models, which indicated function related enrichment with p-value <0.01 using REVIGO. Aggregate size indicated significance levels in the GO term. (C) K-means clustering showing the expression profile of the 1457 AD/AB DEGs in all tissues (SAM, LP-AD, LP-AB, L-B, L-M, and L-T). (D) Overview of expression of AD/AB DEGs in three leaf positions of rosette leaves. Left the number of genes expressed in LEAF samples is indicated (CCT is Tip position, CCM is mid position, CCB is base position), and the number of DEGs (1457) between Adaxial and Abaxial leaf primordia is indicated. Right the histogram indicates the number of commonly expressed genes in the indicated positions from the total number of LEAF genes that are NOT DEGs between ad/abaxial leaf primordia and from the subset of LEAF genes that is Differentially Expressed between leaf ad/abaxial primordia (indicated by rectangular boxes). The expressed genes have FPKM value >1. (E)Co-expression of AD/AB DEGs in SAM, LP-AD, LP-AB, L-B, L-M, and L-T. Grey lines indicate the connection between two gene groups.

During rosette leaf development, in total 476 genes were differentially expressed along the leaf blade, including 161 DEGs between B and M, 375 DEGs between B and T, 54 DEGs between M and T (Figure 2A). We compared gene expression patterns at Top (T), Mid (M) and Base (B) position of developing leaves to better understand whole leaf growth. By K-means clustering method, these 476 DEGs were divided into seven clusters and four models based on their expression trends (Figure 3A). In model one, 219 DEGs (69 DEGs in cluster 1 and 150 DEGs in cluster 7) were expressed at the highest levels in the base. Whereas, in model two, 166 DEGs (76 DEGs in cluster 3 and 90 DEGs in cluster 4) were expressed at the highest levels in the tip. An additional 25 DEGs in cluster 5 and 66 DEGs (43 DEGs in cluster 2 and 23 DEGs in cluster 7) had a pattern with highest or lowest value in the leaf mid, respectively (Table S4).

**Figure 4:**
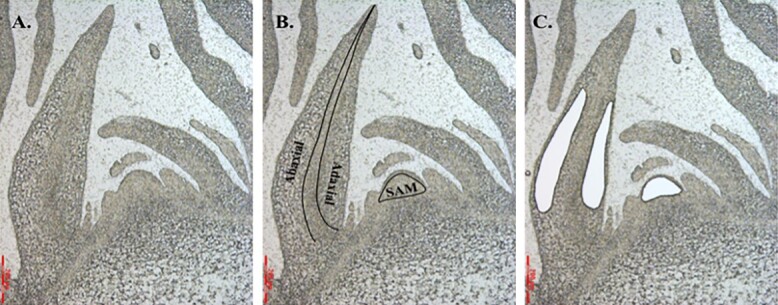
LMD of heading Chinese cabbage CC-Z16 shoot apical meristem and surrounding leaf primordium at the rosette stage (6 weeks after sowing). (A) Representative transverse section of the SAM and leaf primordium. Targeted meristem (SAM) and mesophyll cells (leaf abaxial/adaxial cells without epidermal cells) before (B) and after (C) LMD.

Different categories of gene function are identified for these different expression models during leaf growth (Figure 3B). Model 1 includes genes involved in extracellular region, cell wall biosynthesis and lipid metabolism GO categories, implying the leaf base mainly involves growth related transcripts that accumulate at higher levels in leaf base than tip. For example, *cycp3;1*, promotes cell division at the G2-M duration [24] and the expansin *EXP16* is involved in cell wall expansion [25]. In contrast to this, a relatively high number of genes involved in photosynthesis related processes were highest expressed in the tip of the leaf (model 2), such as photosynthetic electron transport, chlorophyll binding, light signaling, with related genes, including *OHP*, *OHP2* which are functionally lined to photosystem II biogenesis [26], and the early light-induced protein ELIP2 [27]. Other genes include the phototropic-responsive NPH3 family protein DOT3 which produces an aberrant parallel venation pattern in juvenile leaves [28, 29].

In this study, the leaf primordia and rosette leaf were harvested from the same plant. To analyse the regulatory links during leaf development, we explored co-expression of the DEGs between AD and AB, across SAM, AD, AB, LF-T, LF-M, and LF-B samples. After clustering, LP-AD was grouped with SAM, while LP-AB was grouped with positions of expanding rosette leaves (Figure 3C; Table S3). In total, 648 of these 1457 DEGs were expressed in all LEAF (-T, -M, and-B) positions, while 608 were not expressed in any LEAF position. Furthermore, while 85 DEGs of the 1457 were only expressed in LF-B, 12 DEGs only in LF-M and 12 DEGs only in LF-T (Figure 3D; Table S5). These DEGs fell into six expression modules and the genes of each module are listed in Table S3. In cluster 1, 2, and 3, genes were highly expressed in LP-AD, while in cluster 4, 5, and 6, genes were highly expressed in LP-AB (Figure 3C). The well-known leaf polarity related genes of abaxial-axis were distributed in cluster 4, 5, 6, while adaxial-axis genes were only included in cluster 1, characterized by high expression in SAM and AD, with very low or no expression in growing leaves (LEAF). Interestingly only in clusters 1 and 5, genes were expressed in SAM, and either in AD or AB, but not or low expressed in the growing leaf (LEAF). We further analyzed TF genes, which are commonly mentioned during leaf polarity formation, in each cluster. Many TFs in Figure 2D were low or not expressed in expanding rosette leaf, except *YAB2.2*, *KAN2*, and *KAN3*. The *YAB2.2*, *KAN2*, and *KAN3* were included in cluster 4, characterized by high expression in both AB and expanding rosette leaf. It is noteworthy that in Chinese cabbage, there was only one copy for each *KAN2* and *KAN3*. The majority of these leaf-polarity TF genes were found in cluster 1 and cluster 5: *HD-ZIPIII* (*REV, PHV, ATHB15*), *AGO10, ZPR3, BOP1* were in cluster 1 (highly in AD and SAM); *YABBY* (*YAB1, YAB2*), *ARF* (*ARF3, ARF4*) were in cluster 5 (highly in AB and SAM); *YAB1.3, YAB5, KAN1.1, KAN1.2* were in cluster 6 (high in AB only), which indicated that highly expressed TF in AB also displayed high expression levels in LEAF, but highly expressed TFs in AD had low expression levels in LEAF. In addition, some genes connected different clusters, such as transcription factor *PIF4*, cell division gene *CYCD3;1* and *CYCD4;1*, and probably play important roles linking leaf primordia development to leaf growth processes. Genes showing similar expression patterns with these transcription factors and their target genes were identified.

## 3. Discussion

In cabbages, the heading leaves fold around the head and are shielded from the light while and the outer head leaves expose their abaxial side to the light, opposite to normal leaf development, while inner leaves are shielded from the light. This may require a different leaf cellular organization, as the typical palisade cells that have an optimal shape to harvest light, which would not be exposed to light, are redundant. As allelic variation in several of these genes is associated with the typical leafy head phenotype [20], gene expression patterns of the different paralogues of genes with roles in ad/abaxial polarity formation is key to understand the leafy head phenotype of cabbages. Laser dissection of leaf primordia made it possible to compare gene expression profiles between the AD and AB sides and helped capturing the transcriptome changes underlying leaf primordia development. Allelic variation of several genes with roles in leafy head formation play roles in not only ad/ab axial leaf polarity formation, but also in cell division and expansion, miRNA pathways and auxin related pathways. Patterns of gene expression in ad/ab axial sides of leaf primordia can thus not only extent our knowledge about the leaf polarity formation regulatory network, but also provide potential regulator genes for formation of Chinese cabbage leaves and leafy heads.

A connection between SAM and leaf ad/abaxial polarity in *Solanum tubersosum* was found in early surgical experiments that separated a leaf primordium from the SAM resulting in a radially abaxialised leaf. It indicated the SAM could offer a signal to promote adaxial fate while the abaxial fate was established by default ( [30], 1955; [31]). Another study indicates adaxial cell fate plays a vital role in promoting the axillary SAMs development [12]. In Arabidopsis and maize, *HD-ZIPIII* genes are expressed in the SAM and throughout the leaf primordia, confined to the adaxial side of the leaf primordia [15, 32–34]. However, ectopic expression of abaxial specifying factors (*KANADIs* and *YABBY*) has detrimental effects on the SAM, demonstrating that abaxial identity and meristem function are incompatible [35]. In this study, co-expression patterns of DEGs revealed that the SAM clustered with the adaxial side of leaf primordia, while the abaxial leaf primordia clustered with the expanded rosette leaf. Genes highly expressed both in SAM and LP-AD in cluster 1 include *HD-ZIPIII* genes. Genes highly expressed both in LP-AB and LEAF are in cluster 4 including *KAN* and *YAB*. The finding that genes DE between ad/ab axial tissues in leaf primordia are also DE along the leaf blade in expanded leaves corroborates with studies identifying genes selected for their roles in leafy head formation. These studies in both cabbage and Chinese cabbage, identified mutations in *KAN* and additional leaf ad/abaxial polarity genes associated with leafy head development [20, 36]. Initial leaf development of these plants is normal and comparable with non-heading Brassica vegetables. Plants from rosettes, however, after the transition stage, defined by massive changes in gene expression profiles [37] the typical curving of leaves becomes evident, indicating that ad/ab axial polarity genes have important functions during later developing stages in expanded leaves.

Besides the well-known leaf polarity related TFs, many other TF families (195 TFs) were also identified with expression differences between ad/ab axial sides in leaf primordia. For example, several additional *ARF* genes, *ARF5*, *ARF11*, *ARF18*, had similar expression patterns as *ARF3*, with a well-studied role in leaf polarity formation [38]. Also *GATA5* was preferentially expressed in the abaxial cells of Chinese cabbage leaf primordia. The family of GATA-binding zinc finger transcription factors, plays diverse functions in a wide array of biological processes, like *GATA2* mediating the crosstalk between BR- and light-signaling pathways [39] and *GATA5* involved in xylem vessel formation [40], with no reports on its involvement in leaf polarity formation. In this study, the abaxial cells of Chinese cabbage leaf primordia where *GATA5* was preferentially expressed represent the tissue in which the vessels will form upon leaf maturation. Most of the multiple copies of leaf polarity related TF genes in Chinese cabbage have similar expression patterns as *Arabidopsis* genes, such as *ARF3.1* and *ARF3.2*, all high expressed in AB. When multiple gene copies show similar expression patterns, one assumption is that these copies all contribute to leaf polarity. In contrast, some paralogues showed differences in transcript abundance. For example, *ARF4.1* is highly expressed in AB, but *ARF4.2* is not expressed in SAM, LP or leaf. In addition, some of these TFs show differences in expression patterns between Chinese cabbage and *Arabidopsis*. In *Brassica*, some ad/abaxial genes have been reported to be related to leafy head formation have been reported, such as transcription factor *ARF3.1*, *ARF4.1*, *KAN2.1* [20], *Aux1*, and *Aux2* [41], *BcpLH* [42], *TCP* [43], *BrpSPL9* [44], auxin transport genes *BrLAX*, *BrPIN*, and *BrPGP* [45]. These genes or genes from the same family were identified as DEGs between AD and AB leaf primordia in this study, linking again formation of ad/ab axial polarity to leafy head formation, where leaves curve inward and outer head leaves expose their abaxial sites to the light.

We compared the DEGs between ad/ab axial primordia in this study with two studies that also compared gene expression between palisade and spongy mesophyll cells. Gou et al. (2022) prepared protoplasts from young leaves of Chinese cabbage at the rosette stage for single-cell RNA-seq (scRNA-seq) and selected 433 and 510 potential DEGs with increased expression in palisade mesophyl cells (AD) and spongy mesophyl cells (AB). Xia et al. [46] established the single-cell spatial transcriptome technique (scStereo-seq) in *Arabidopsis* rosette leaves and identified transcriptional differences between cell subtypes, including palisade mesophyll cells and spongy mesophyll cells. When comparing these two studies with our work, we found that only a few ad/ab DEGS overlapped between these studies (Figure S1; Table S6). A possible explanation is that both studies [46, 47] used expanded rosette leaves of Chinese cabbage and *Arabidopsis*, respectively, for their analyses, with differentiated palisade- and spongy tissue, while our study used leaf primordia, with still undifferentiated palisade and spongy tissue.

The aim of this study was to describe gene expression profiles for both the SAM and leaf primordia and growing rosette leaves in Chinese cabbage. This resulted in a broad range of genes differentially expressed between ad/ab axial primordial cells with likely roles in leaf polarity formation. We clustered these differentially expressed ad/ab axial leaf primordia genes based on their co-expression in also SAM and rosette leaves. We hypothesize that the ad/ab axial genes that were also expressed in rosette leaves represent interesting candidates for their role in leafy head formation. In addition, we generated a list of tissue specific genes, which are of interest as potential cell-type markers, allowing exploration for further functional identification of leaf tissue cells in Chinese cabbage. In conclusion, combined analyses of gene expression profiles from Chinese cabbage micro dissected AD- and AB-leaf primordia, SAM and rosette leaves, resulted in a catalogue of genes that shed light on early rosette leaf development and is the basis for further studies towards the genetic mechanisms of leafy head formation that is associated with allelic variation in leaf ad/ab axial genes.

## 4. Materials and methods

### 4.1. Plant materials

The heading Chinese cabbage (CC-Z16) seeds were sown in July 2017. Seeds were germinated in seeding soil for one week and then plants were transplanted into Ø17 cm pots in the greenhouse and grown under long day conditions at Unifarm, Wageningen University & Research (51^°^59′11′′N latitude, 05^°^39′52′′E longitude).

### 4.2. Sample collection and RNA amplification

#### 4.2.1. Collection of cells by laser capture microdissection (LCM) and subsequent RNA amplification

At rosette stage (week 4 after transplanting; 6 weeks after sowing), shoot apical meristems with surrounding leaf primordia were harvested of Chinese cabbage CC-Z16 with three biological replicates. Leaves around the SAM were removed until only a few leaf primordia were left. Tissues containing SAM and few leaf primordia were cut and immediately placed in Farmer solution (75% ethanol, 25% acetic acid). Samples were kept overnight in fixation solution at 4°C and dehydrated in increasing concentrations of ethanol (30%, 70%, 96%, and 3×100%) for 20 minutes per step. Steedman wax was mixed by 90% polyethylene glycol 400 distearate and 10% 1-hexadecanol at 65°C. The tissues were then first replaced with ethanol/Steedman wax (1:1) mixture for 5 hours, followed by three times incubation in 100% Steedman wax in 24 hours at 37°C. Finally, the tissues were oriented in the molds containing Steedman wax and sectioned using a microtome at 9 μm. The tissue section was floated in RNAse free water containing 40 mM DTT on a UV-treated 4 μm PEN-membrane slide (Leica). Slides were washed in 100% ethanol for 3 minutes to remove the Steedman wax and air-dried for laser capture microdissection (LCM). Cells from shoot meristem, leaf abaxial and -adaxial tissues were harvested from the slides using a Leica LMD7000 laser microdissection microscope and cells were collected into the cap of 0.5 ml Eppendorf tube with 70 μl RLT buffer containing 40 mM DTT, respectively (Figure 4). In total ~ 4000 cells were collected per sample. RNA was isolated using the RNeasy Micro kit (Qiagen) from each cell type and amplified using the SMART-Seq v4 Ultra Low Input RNA Kit (Clonetech), following the manufacturer's instructions. Three biological replicates were used for the RNA-seq experiments.

#### 4.2.2. Rosette leaf sampling from three leaf positions and RNA amplification

Leaf samples were harvested from three positions: tip (T), middle (M) and base (B) of the expanding rosette leaf for heading cabbage genotype CC-Z16 at week 6 after sowing (Figure S2). Leaf cytological observations were performed at the same time for these three positions. Three biological replicates (= 3 different plants) were used for RNA isolation and RNAseq. The total RNA was extracted using Plant RNeasy Mini kit (QIAGEN) for RNAseq transcriptome analysis. The RNA isolated from cells (SAM, adaxial-AD and abaxial-AB) and rosette leaf tissues (T, M, and B) were subjected to transcriptome sequencing (RNA-seq).

### 4.3. RNA sequencing and data analysis

The RNA isolated from cells (SAM, AD and AB) were subjected to transcriptome sequencing (RNA-seq). RNA quality and quantity were measured using the Nanodrop 2500 (Thermo Fisher Scientific, US). RNA-seq libraries were constructed and sequenced. *B. rapa* cv. *Chiifu* (a leafy vegetable inbred line) genome sequence (V3.0) was used as the reference genome.

Transcript levels were calculated in FPKM value by RSEM software (http://deweylab.biostat.wisc.edu/rsem/) and expressed genes were defined as FPKM >1. All gene transcript abundance data were used for principal coordinated analysis (PCA) to check the dominant modes of variation in different samples. The highly expressed genes across the SAM, AD, AB tissues, and LF-B, -M, -T leaf positions were only expressed in a single tissue or position with cutoff value of 1 FPKM. For each tissue or position, FPKM for three biological replicates need to meet the screening criteria. Differentially expressed genes (DEGs) were defined as fold change (FC) > = 2 and *P* value <0.05 by using the tool DESeq2 method (http://bioconductor.org/packages/stats/bioc/DESeq2/).

The identified DEGs were clustered using K-means method. Gene ontology (GO) terms (http://www.geneontology.org/) and Kyoto Encyclopedia of Genes and Genomes (KEGG) pathways (http://www.genome.jp/kegg/) enrichment analysis were performed for all transcript genes and DEGs. Furthermore, co-expression network analysis was performed and gene pairs with at least spearman correlation coefficient of 0.9 and *P* < 0.01 were considered. Cluster method “complete” distance method “euclidean”.

## Supplementary Material

Web_Material_uhae059

## Data Availability

The data underlying this article are available in the article and in its online supplementary material. The raw sequence data reported in this paper have been deposited in the Genome Sequence Archive (Genomics, Proteomics & Bioinformatics 2021) in National Genomics Data Center (Nucleic Acids Res 2022), China National Center for Bioinformation / Beijing Institute of Genomics, Chinese Academy of Sciences (GSA: CRA013309) that are publicly accessible at https://ngdc.cncb.ac.cn/gsa.
