# Supplementary material for: Transcriptomic analyses to summarize gene expression patterns that occur during leaf initiation of Chinese cabbage
Source: Hortic Res. 2024 Feb 28;11(4):uhae059. doi: 10.1093/hr/uhae059 (PMC11059812; doi:10.1093/hr/uhae059)
Supplement: Web_Material_uhae059 [file web_material_uhae059.zip › Table S7.pdf]

Brassica ID	Pathway	Sub-pathway	AT ID	Gene nume	
BraA01g015820.3C	redox	redox.dismutases and catalases	AT4G25100.1	ATFSD1,FSD1	Fe superoxide dismutase 1
BraA01g019200.3C	RNA	RNA.regulation of transcription.HB,Homeobox transcription factor family	AT4G17460.1	HAT1	Homeobox-leucine zipper protein 4 (HB-4) / HD-ZIP protein
BraA01g023890.3C	misc_otherPhosphate	misc.peroxidases	AT3G49120.1	DAAR1	peroxidase CB
BraA01g041170.3C	not_assigned	not assigned.unknown	AT3G08030.1		Protein of unknown function, DUF642
BraA02g007960.3C	#N/A	#N/A	#N/A	#N/A	#N/A
BraA02g016000.3C	hormone_metabolism	hormone metabolism.jasmonate.synthesis-degradation.lipoxygenase	AT3G45140.1	ATLOX2,LOX2	lipoxygenase 2
BraA02g017840.3C	cell_wall	cell wall.degradation.pectate lyases and polygalacturonases	AT1G67750.1		Pectate lyase family protein
BraA02g024180.3C	misc_otherPhosphate	misc.O-methyl transferases	AT1G77520.1		O-methyltransferase family protein
BraA02g026160.3C	not_assigned	not assigned.unknown	AT1G80130.1		Tetratricopeptide repeat (TPR)-like superfamily protein
BraA02g041220.3C	not_assigned	not assigned.unknown	AT5G26280.1		TRAF-like family protein
BraA02g041570.3C	stress	stress.abiotic.heat	AT5G25530.1		DNAJ heat shock family protein
BraA02g042050.3C	metal_handling	metal handling.binding, chelation and storage	AT5G24580.1		Heavy metal transport/detoxification superfamily protein
BraA03g003410.3C	protein	protein.degradation.serine protease	AT5G08260.1	scpl35	serine carboxypeptidase-like 35
BraA03g006120.3C	not_assigned	not assigned.unknown	AT5G14090.1		
BraA03g009940.3C	stress	stress.abiotic.heat	AT5G20970.1		HSP20-like chaperones superfamily protein
BraA03g023540.3C	cell	cell.cycle	AT2G45080.1	cycp3;1	cyclin p3;1
BraA03g031580.3C	misc_otherPhosphate	misc.GDSL-motif lipase	AT3G04290.1	ATLTL1,LTL1	Li-tolerant lipase 1
BraA03g050250.3C	nucleotide_metabolism	nucleotide metabolism.salvage.phosphoribosyltransferases.aprt	AT4G22570.1	APT3	adenine phosphoribosyl transferase 3
BraA03g053010.3C	#N/A	#N/A	#N/A	#N/A	#N/A
BraA03g059190.3C	redox	redox.dismutases and catalases	AT4G35090.1	CAT2	catalase 2
BraA04g004160.3C	not_assigned	not assigned.unknown	AT3G56220.1		transcription regulators
BraA04g008690.3C	cell_wall	cell wall.cellulose synthesis	AT3G56000.1	ATCSLA14,CSLA14	cellulose synthase like A14
BraA04g024760.3C	RNA	RNA.regulation of transcription.HB,Homeobox transcription factor family	AT2G34710.1	ATHB-14,ATHB14,PHB,PHB-1D	Homeobox-leucine zipper family protein / lipid-binding START domain-containing protein
BraA05g004850.3C	RNA	RNA.regulation of transcription.C2C2(Zn) GATA transcription factor family	AT2G45050.1	GATA2	GATA transcription factor 2
BraA05g025150.3C	#N/A	#N/A	#N/A	#N/A	#N/A
BraA06g009240.3C	amino_acid_metabolism	amino acid metabolism.misc	AT1G12420.1	ACR8	ACT domain repeat 8
BraA06g026370.3C	not_assigned	not assigned.unknown	AT5G62720.2		Integral membrane HPP family protein
BraA06g043050.3C	#N/A	#N/A	#N/A	#N/A	#N/A
BraA07g013840.3C	#N/A	#N/A	AT1G24020.1	MLP423	MLP-like protein 423
BraA07g028740.3C	major_CHOmetabolism	major CHO metabolism.degradation.sucrose.Susy	AT1G73370.1	ATSUS6,SUS6	sucrose synthase 6
BraA07g030710.3C	misc_otherPhosphate	misc.gluco-, galacto- and mannosidases.alpha-galactosidase	AT1G68560.1	ATXYL1,TRG1,XYL1	alpha-xylosidase 1
BraA07g031010.3C	#N/A	#N/A	#N/A	#N/A	#N/A
BraA07g033670.3C	transport	transport.peptides and oligopeptides	AT1G68570.1		Major facilitator superfamily protein
BraA07g035810.3C	RNA	RNA.regulation of transcription.bHLH,Basic Helix-Loop-Helix family	AT1G71200.1		basic helix-loop-helix (bHLH) DNA-binding superfamily protein
BraA08g018790.3C	not_assigned	not assigned.unknown	AT4G29020.1		glycine-rich protein
BraA08g030620.3C	misc_otherPhosphate	misc.cytochrome P450	AT1G13080.1	CYP71B2	cytochrome P450, family 71, subfamily B, polypeptide 2
BraA09g004810.3C	amino_acid_metabolism	amino acid metabolism.synthesis.serine-glycine-cysteine group.cysteine.OASTL	AT5G28030.1	DES1	L-cysteine desulfhydrase 1
BraA09g028460.3C	stress	stress.biotic	AT4G09950.1		P-loop containing nucleoside triphosphate hydrolases superfamily protein
BraA09g040190.3C	RNA	RNA.RNA binding	AT1G22910.1		RNA-binding (RRM/RBD/RNP motifs) family protein
BraA09g065870.3C	signalling	signalling.light	AT1G02340.1	FBI1,HFR1,REP1,RSF1	basic helix-loop-helix (bHLH) DNA-binding superfamily protein
BraA10g004540.3C	stress	stress.biotic	AT1G05760.1	RTM1	Mannose-binding lectin superfamily protein
BraA10g007150.3C	#N/A	#N/A	#N/A	#N/A	#N/A
BraA10g011780.3C	not_assigned	not assigned.unknown	AT5G53420.1		CCT motif family protein
BraA10g019040.3C	development	development.unspecified	AT5G22290.1	anac089,NAC089	NAC domain containing protein 89
BraA10g022320.3C	protein	protein.degradation.AAA type	AT5G17760.1		P-loop containing nucleoside triphosphate hydrolases superfamily protein
BraAnng005410.3C	not_assigned	not assigned.unknown	AT3G01670.1		
BraA07g021740.3C	secondary_metabolism	secondary metabolism.phenylpropanoids	AT5G67150.1		HXXXD-type acyl-transferase family protein
BraA07g009310.3C	metal_handling	metal handling	AT3G23800.1	SBP3	selenium-binding protein 3
BraA01g023560.3C	hormone_metabolism	hormone metabolism.ethylene.synthesis-degradation	AT3G49630.1		2-oxoglutarate (2OG) and Fe(II)-dependent oxygenase superfamily protein
