# Supplementary figures and images for: Transcriptomic analyses to summarize gene expression patterns that occur during leaf initiation of Chinese cabbage
Source: Hortic Res. 2024 Feb 28;11(4):uhae059. doi: 10.1093/hr/uhae059 (PMC11059812; doi:10.1093/hr/uhae059)

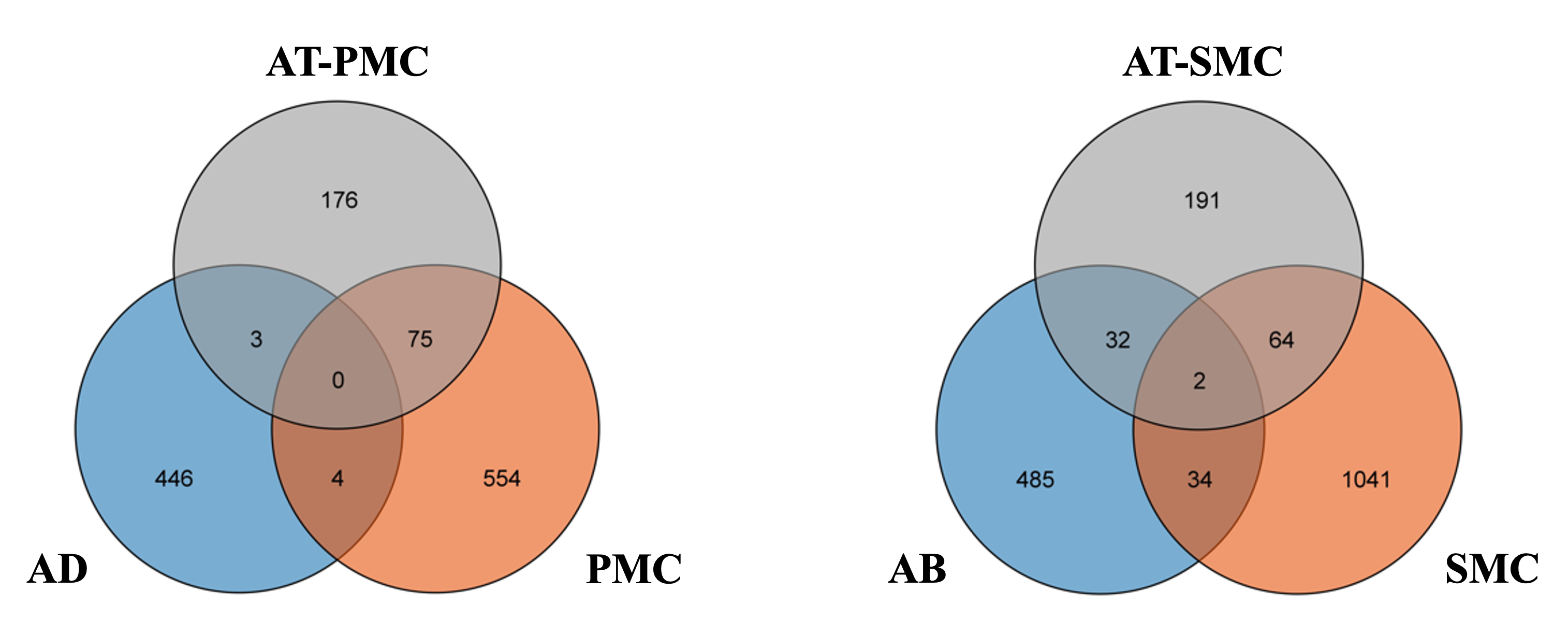

Supplement: Web_Material_uhae059 [file web_material_uhae059.zip › Figure S1.png]

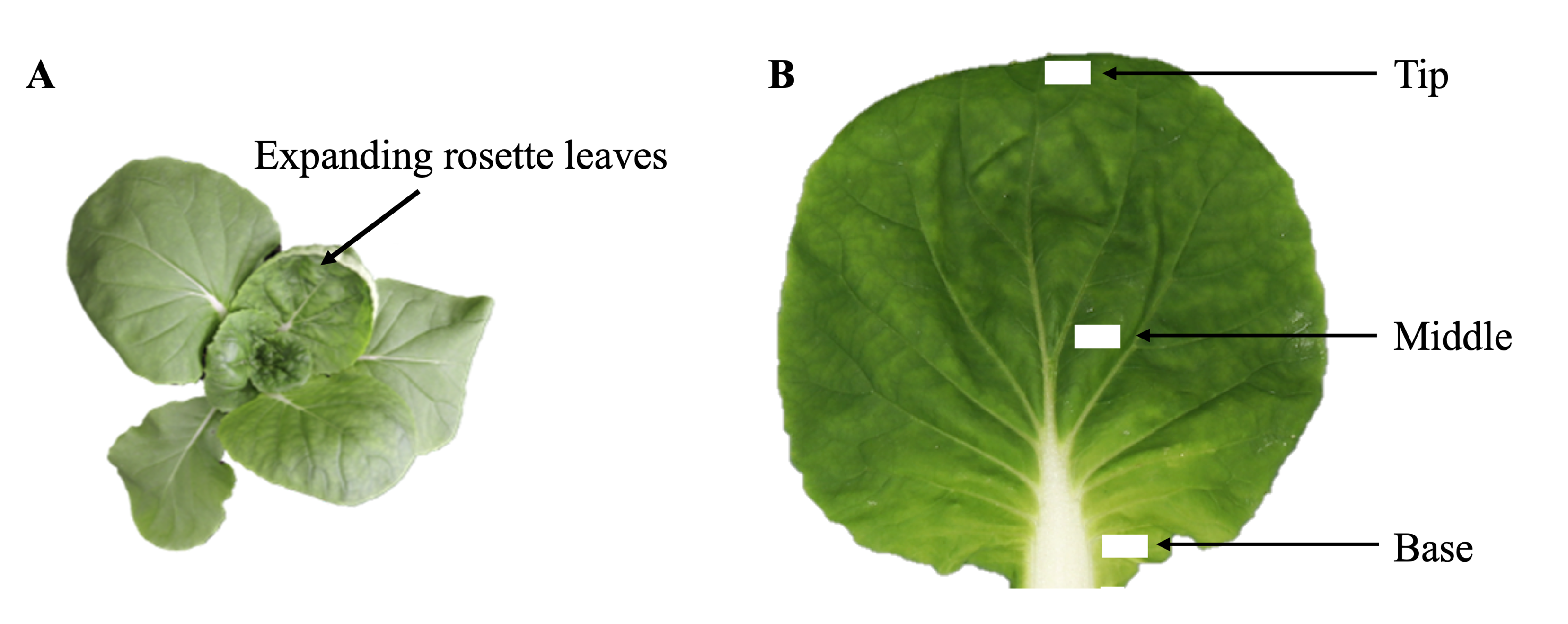

Supplement: Web_Material_uhae059 [file web_material_uhae059.zip › Figure S2.png]
